# Physical methods for synchronization of a human production cell line

**DOI:** 10.1186/1753-6561-5-S8-P49

**Published:** 2011-11-22

**Authors:** Oscar Platas Barradas, Uwe Jandt, Ralf Hass, Cornelia Kasper, Volker Sandig, Ralf Pörtner, An-Ping Zeng

**Affiliations:** 1 Institute of Bioprocess and Biosystems Engineering, Hamburg University of Technology, Hamburg, 21073, Germany; 2 Gynecology and Obstetrics. Medical University Hannover, Hannover, 30625, Germany; 3 Institute for Technical Chemistry, Leibniz University Hannover, Hannover, 30167, Germany; 4 ProBioGen AG, Berlin, 13086, Germany

## Background

The study of central metabolism and the interaction of its dynamics during growth, product formation and cell division are key tasks to decode the complex metabolic network of mammalian cells. For this purpose, not only the quantitative determination of key cellular molecules is necessary, but also the variation of their expression rates in time, e.g. cell cycle dependent gene expression. Thus, synchronization of cultured cells is a requisite for almost any attempt to elucidate these time dependent cellular processes. Synchronous cell growth can help to gain deeper insight into dynamics of cellular metabolism.

In our work, physical methods for synchronization of the human production cell line AGE1.HN (ProBioGen AG) are experimentally tested. Cell-size distribution, DNA-content and the number of synchronous divisions are used for comparison of the methods.

According to our results, the enrichment of an AGE1.HN cell population within a cell cycle phase is possible. Currently, the increase of cell yield and the improvement of conditions for cell-growth resumption after synchronization are being studied.

## Synchronization methods

Synchronization of cells can be defined as the enrichment of cells within a certain phase of the cell cycle. Many authors have pointed to the importance of further synchronous growth or even a narrow cell size distribution. Thus, a synchronized culture is one in which cells of similar age progress as a cohort through the division cycle [1].

Physical and chemical methods for cell synchronization are well described in literature. The choice depends not only on the cell type (suspension, adherent, growth medium, etc.), but also on the desired yield of cells and the degree of synchrony. For the analysis of cell-cycle dependent metabolic processes, the method of choice should not alter the rate of cellular reactions as they occur normally. The following physical methods are part of this study: **(1) Temperature reduction:** cell growth can be slowed down by means of a reduction of temperature during culture [2], which allows for enrichment of cell populations within the G_1_ and early S-phase. Since yields obtained by using this method are low, temperature cycles can be used for yield improvement. **(2) Gradient centrifugation:** cells can be separated according to their density in a gradient by centrifugation. A sucrose gradient can be used for this purpose, with the advantage of being a simple and less expensive procedure. **(3) Counterflow centrifugal elutriation:** cells are separated in a centrifugal field; at the same time, a fluid in counterflow is used to separate the cells according to their mass within the centrifugal field. Depending on the mass of the cell, cell populations can be eluted out of the system by increasing the flow rate of the fluid.

## Materials and methods

**(1) Temperature reduction:** shake flasks were inoculated with 1·10^6^ cells mL^-1^ and cultivated at 37 °C and 5 % CO_2_. After 24 h the flasks were set at a reduced temperature. Sampling was performed at least every 24 h. Samples were treated for flow-cytometry analysis. For yield improvement a repeated-batch strategy with temperature cycles (37 °C → 30 °C, 28 °C) was performed in a controlled bioreactor (1 L, pH = 7.15, DO = 25 % air sat.).

**(2) Gradient centrifugation:** discontinuous sucrose gradients were prepared in 50 mL centrifuge tubes. A first gradient considered 5 to 60 % (w/w) of sucrose in culture medium. In order to avoid undesired mixing of the gradient layers during layer addition, the tubes were set every time at -80°C after each new addition. Phenol red was used for visual identification of the layers. A second gradient was produced (20 – 50 % w/w sucrose), which approaches to the density range of AGE1.hn cells. 1.5·10^8^ cells were centrifuged and added to the top of the gradient. Centrifugation was performed at 230 *g* for 10 min. After centrifugation samples from the gradient were washed in phosphate saline solution (PBS) and analyzed for cell-size distribution (Z2 Particle Counter, Beckman Coulter, Germany).

**(3) Counterflow centrifugal elutriation:** 7·10^8^ cells were centrifuged and resuspended in 5 mL PBS. This volume was inserted under sterile conditions into an elutriator (Beckman Coulter) and pumped at a minimal velocity into the separation chamber. Pumping rate of sterile PBS was increased stepwise in order to elute fractions of cells. Nine fractions were collected. The size distribution of the fractions and their DNA-content (flow cytometry) were analyzed. All fractions were resuspended in fresh medium and cultivated in an incubator.

## Results and conclusions

Pre-experiments for **temperature** reduction had shown previously an increase of up to 80% of S-Phase DNA-content during shake-flask culture at 30 °C. Further temperature reduction (28 °C) was needed during repeated batch cultivation with temperature cycles for cell growth arrest. A viability decrease after temperature resumption (37°C) was observed after 50 h at 28 °C and might be attributed to apoptosis. Duration of the temperature cycles is being currently studied.

High viability was observed in all samples after **gradient centrifugation**. Cell size distribution was reduced for one sample to almost 80 % of cells between 12.5 and 15 µm. Gradient can be reduced in order to sharpen the separation of the cells. Further culture of a cell population and cell cycle analysis are needed to show the feasibility of this method.

**Figure 1:**
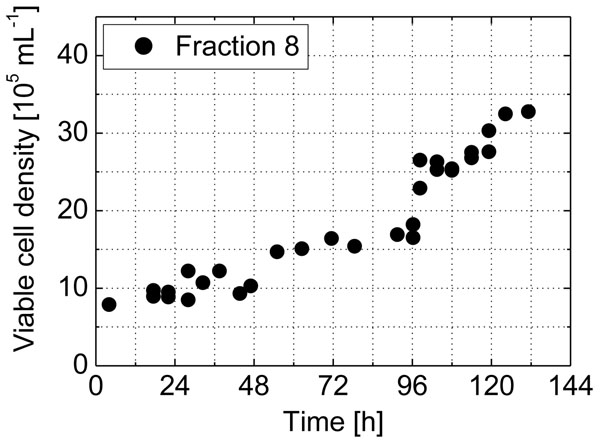
6-day culture of ELU8. Further culture was possible without noticeable perturbation of cell metabolism.

By using the **Counterflow Centrifugal Elutriation**, cells enriched in different phases of the cell cycle were obtained. The cell-cycle analysis and the growth curve of one of the fractions (ELU 8) are presented in Table 1 and Figure 1.

**Table 1:** Flow cytometry analysis of one elutriated fraction (ELU8).

Cell cycle phase	Non-Sync.	Elu8
sub G_1_	1,5	0,6
G_0_/G_1_	61,6	5,9
S	17,8	20,8
G_2_/M	14,2	**52,6**

An increase of more than 35 % of cells in the G2/M-Phase was observed for this fraction.

Funding by the BMBF, Grand Nr. 0315275A is gratefully acknowledged.
